# Analyzing Patient Trajectories With Artificial Intelligence

**DOI:** 10.2196/29812

**Published:** 2021-12-03

**Authors:** Ahmed Allam, Stefan Feuerriegel, Michael Rebhan, Michael Krauthammer

**Affiliations:** 1 Department of Quantitative Biomedicine University of Zurich Zurich Switzerland; 2 Biomedical Informatics University Hospital of Zurich Zurich Switzerland; 3 Department of Management, Technology, and Economics ETH Zurich Zurich Switzerland; 4 ETH Artificial Intelligence Center ETH Zurich Zurich Switzerland; 5 Ludwig Maximilian University of Munich Munich Germany; 6 Yale Center for Medical Informatics Yale University School of Medicine New Haven, CT United States

**Keywords:** patient trajectories, longitudinal data, digital medicine, artificial intelligence, machine learning

## Abstract

In digital medicine, patient data typically record health events over time (eg, through electronic health records, wearables, or other sensing technologies) and thus form unique patient trajectories. Patient trajectories are highly predictive of the future course of diseases and therefore facilitate effective care. However, digital medicine often uses only limited patient data, consisting of health events from only a single or small number of time points while ignoring additional information encoded in patient trajectories. To analyze such rich longitudinal data, new artificial intelligence (AI) solutions are needed. In this paper, we provide an overview of the recent efforts to develop trajectory-aware AI solutions and provide suggestions for future directions. Specifically, we examine the implications for developing disease models from patient trajectories along the typical workflow in AI: problem definition, data processing, modeling, evaluation, and interpretation. We conclude with a discussion of how such AI solutions will allow the field to build robust models for personalized risk scoring, subtyping, and disease pathway discovery.

## Introduction

Digital medicine facilitates broad access to large volumes of patient data, typically through recordings of health events over time. For example, electronic health records store the history of a patient’s diagnoses, medications, laboratory values, and treatment plans [1-3]. Wearables collect granular sensor measurements of various neurophysiological body functions over time [4-6]. Intensive care units (ICUs) monitor disease progression via continuous physiological measurements (eg, electrocardiograms) [7-10]. As a result, patient data in digital medicine are regularly of longitudinal form (ie, consisting of health events from multiple time points) and thus form *patient trajectories*.

**Figure 1:**
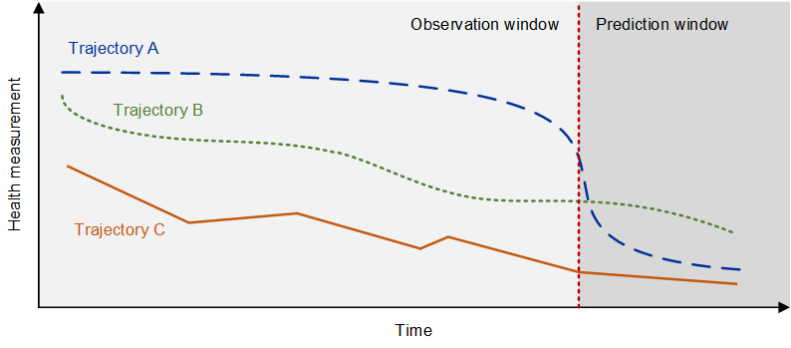
Analyzing patient trajectories with artificial intelligence in digital medicine.

Analyzing patient trajectories provides opportunities for more effective care in digital medicine [2,7,11]. Patient trajectories encode rich information on the history of health states that are also predictive of the future course of a disease (eg, individualized differences in disease progression or responsiveness to medications) [9,10,12]. As such, it is possible to construct patient trajectories that capture the entire disease course and characterize the many possible disease progression patterns, such as recurrent, stable, or rapidly deteriorating disease states (Figure 1). Hence, modeling the patient trajectories allows one to build robust models of diseases that capture disease dynamics seen in patient trajectories. Here, we replace disease models with data from only a single or a small number of time points by disease models that account for the longitudinal nature of patient trajectories, thus offering vast potential for digital medicine.

Several studies have previously introduced artificial intelligence (AI) in medicine for practitioners [13,14]. Some studies review potential medical applications that could benefit from AI [15,16], whereas others review specific methods (eg, deep learning [17,18]) or specific data types (eg, electronic health records [18] and medical images [19]). Some studies suggest reporting guidelines [20,21] or discuss the integration of AI into medical practice [22]. Our research contributes to the literature by discussing AI-powered digital medicine based on patient trajectories.

Patient trajectories refer to time-resolved representations of patient health events across multiple time points (eg, hospitalization or treatment events, sensor measurements from wearables, and physiological measurements from ICUs). AI-powered analysis of patient trajectories allows for an assessment of the heterogeneity of patient disease courses. In Figure 1, trajectory A predicts a sudden but sharp decline in a health state, whereas trajectories B and C depict 2 types of progressively declining disease states. Analyzing trajectory-like representations of patient data can generate new insights for better care in digital medicine.

To unlock the value of patient trajectories for digital medicine, there is a need for new AI solutions that can deal with time-resolved sequential data consisting of multiple health events. Although many models from the area of AI have become standard in digital medicine (eg, deep learning [18]), a naïve application of such models might not be effective when modeling the longitudinal nature of patient trajectories. Instead, this requires customized approaches. For example, in a study by Alaa et al [23], a direct application of deep learning has been found to be outperformed in terms of both predictive accuracy and interpretability when one instead uses a carefully engineered sequential model (ie, referred to as Hawkes process) that treats the time between medical events as informative for the course of the disease. On the basis of this background, we discuss challenges and solutions for AI that are unique to analyzing patient trajectories. Specifically, we examine the implications for developing disease models from the patient trajectories along the typical workflow in AI: (1) problem definition, (2) data processing, (3) modeling, (4) evaluation, and (5) interpretation, as detailed in the following section.

## Applying AI to Patient Trajectories

### Problem Definition

Applying AI to patient trajectories is relevant for different objectives in digital medicine (Table 1). One objective is *risk scoring* [3,23,24], where patient trajectories are leveraged to predict patient outcomes. Here, the rationale is that the predictions based on health measurements from patient trajectories with multiple time points have greater predictive power than the predictions from those with a single or a few time points. For instance, risk scoring in ICUs becomes more accurate when traditional scores (eg, Acute Physiology and Chronic Health Evaluation II and Simplified Acute Physiology Score) are replaced with AI-based predictions incorporating data from patient trajectories [9,10,25]. Similarly, for cardiovascular diseases, existing risk scores (eg, the Framingham risk score that predicts the 10-year risk of developing coronary heart disease) become more accurate when replaced by AI solutions that work with longitudinal patient data [12]. These examples show that the underlying patient trajectory provides rich, granular insights into the disease dynamics that can then be captured by AI solutions for trajectory analysis. Therefore, additional patient information, such as past medications, comorbidities, or other risk factors, can be considered. For instance, in the context of cardiovascular diseases, it might be informative for risk scoring to analyze the patient’s past journey, which comprises whether the patients have been prescribed nicotine replacements and *when* (eg, only recently or several years ago). Different patient outcomes including mortality, hospital readmission, hospital length of stay, disease onset, disease severity, or adverse drug reactions can be of interest in risk scoring. The risk score can then inform treatment planning (or in general, assess the patients’ needs). In addition, AI solutions can further generate insights for defining (early) disease states.

**Table 1:** Overview of different objectives in artificial intelligence–based trajectory analysis.

Objective	Description	Examples	Selected references
Risk scoring	The objective is to estimate the likelihood of future health outcomes (eg, mortality, readmission, and adverse drug reactions)	Predict the 10-year risk of developing coronary heart disease for patients as in the Framingham risk score Predict the need for an intensive care unit in an emergency ward through measurements from wearables	[3,17,23,26-32]
Subtyping	The objective is to cluster the patient cohort into different disease dynamics (ie, subtyping) while accounting for the longitudinal form of patient trajectories	Cluster disease progressions into “recurrent course” and “progressive decline”	[26]
Pathway discovery	The objective is to detect clinically meaningful subpatterns in patient trajectories	Identify frequent patterns in patient trajectories that are indicative of disease onset	[1,33,34]

Here, we see several paths for risk scoring based on patient trajectories. First, to ensure high accuracy, AI-based risk scores must be tailored to each patient outcome while considering the desired forecast horizon and the patient cohort. So far, there are several studies that showcase the successful use of AI-based risk scores [35,36]; however, there is a need to develop other risk scores, especially for the settings in which AI-based risk scores are scarce or not yet available (eg, predicting the transition from prediabetes to diabetes or predicting specific adverse reactions to medication). Second, the AI-based risk scores will need to be integrated more extensively in clinical practice. Third, the risk scores should be extensively combined with approaches for explainability or interpretability, which allow the derivation of clinically relevant insights from patient trajectory data (eg, which information in a patient trajectory is a risk factor). Finally, if one includes data on treatments in the risk scoring model, one may infer the expected individualized treatment effect and eventually guide the treatment selection [37-42]. Here, we see further potential to transition from a purely predictive approach (ie, what is the expected risk level) to a prescriptive approach (ie, what treatment do we expect to reach a desired patient outcome). However, many AI solutions for estimating individualized treatment effects from patient trajectories have recently emerged [37-42] without being tailored to specific disease settings and patient cohorts. Therefore, further research at the interface to digital medicine should be a priority that will eventually yield effective and robust implementations for clinical practice.

Another objective of AI in digital medicine is *subtyping*, where AI can understand the heterogeneity observed in patient trajectories and identify the corresponding digital markers. A simple approach is to cluster the different patient trajectories (ie, subtyping) to match patients with similar disease dynamics, clinical pathways, or care patterns [26]. As a practical benefit, subtyping can support clinical tasks related to cohort building and, for instance, can provide patient stratification (eg, to define a cluster of patient trajectories that serves as an inclusion criterion for a clinical trial). However, subtyping requires a suitable notion of patient similarity, which can be challenging to define mathematically because of the longitudinal form of patient trajectories. Thus, it is crucial not only to cluster risk factors at baseline but also to find mathematical approaches that account for the temporal nature of the trajectories (ie, time-series clustering). This allows clinical practice to identify subgroups or subtypes based on the underlying disease dynamics (eg, to distinguish subgroups with a recurrent course vs a progressive decline). We expect an added value from comparing different subtyping approaches in terms of their relative strengths (eg, generated insights) for future research. On this basis, digital medicine could develop a principled procedure for defining *patient trajectory similarity* in the context of subtyping.

A related objective is *pathway discovery***,** where patterns in patient trajectories should be detected [1,33]. For instance, 1 application analyzes time series with laboratory measurements from patients with hepatitis B and C to discover frequent patterns indicative of liver damage [34]. This application can help to understand the underlying course of diseases and identify short- and long-term patterns (ie, motifs) in patient trajectories.

Depending on the objective and explicit assumptions, implications arise regarding the AI workflow and thus highlight the importance of selecting an appropriate modeling strategy. Additional details are provided in the following sections.

### Data Processing

A fundamental question is concerned with data collection as this defines how time is encoded in the data. The example of nicotine replacement suggests that we should know whether such a medical event was recent or several years ago. This illustrates where we are now: when we have longitudinal data, it is often a matter of whether a medical event happened *recently* or *a while back*. Thus, the underlying time (or the underlying time lag) must be carefully considered to capture the longitudinal dimension of patient trajectories correctly. This is currently a challenge when considering the survey designs (eg, for risk scores). For example, one survey may ask a patient whether an event occurred last month or earlier, whereas another survey may ask to consider events that occurred within the last 12 months or earlier. As such, the meaning of *recent* may be inconsistent across survey designs. As a way forward, it will be necessary to develop a more consistent understanding, ideally involving data collection that considers precise time stamps (eg, by leveraging electronic health records).

**Figure 2:**
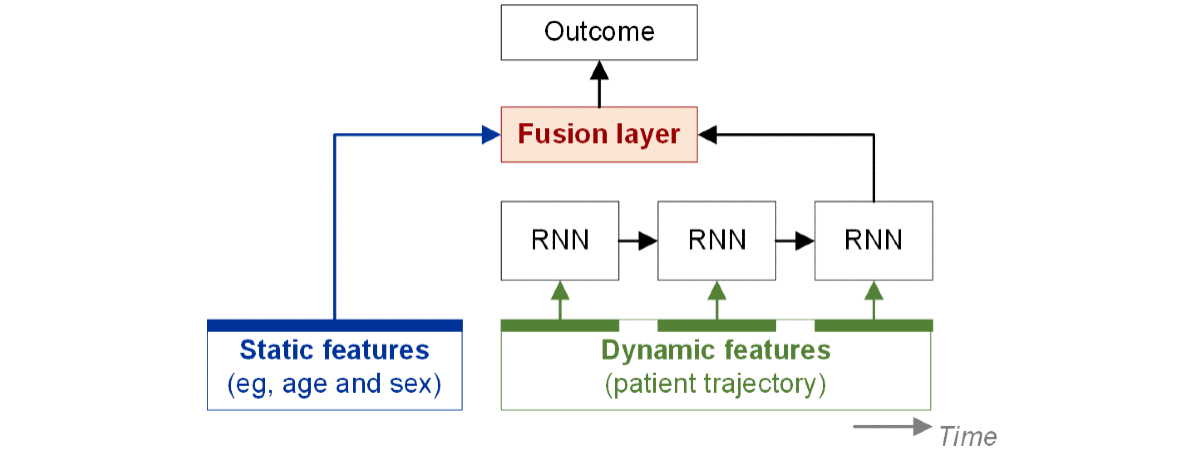
Example of artificial intelligence–based trajectory analysis. RNN: recurrent neural network.

The AI-based trajectory analysis often combines data from patient trajectories and baseline variables that describe risk factors at the patient level (eg, sociodemographic, genomic data, or multimodal data). To combine sequential and static baseline data, tailored AI solutions will need to be developed [27]. Figure 2 shows an example of this approach. The literature shows larger variability regarding the modeling approaches; hence, further evaluations are needed to inform an effective approach.

Figure 2 shows an AI-based trajectory analysis in which a fusion layer combines the static (eg, age or sex) and dynamic features (eg, health measurements over time). The dynamic features have a longitudinal form and are processed by a sequential model (here, a recurrent neural network [RNN]).

Data from patient trajectories are often complex and high-dimensional, which presents difficulties to humans and AI solutions for making accurate inferences. As a remedy, one can apply approaches that map patient trajectories onto a lower-dimensional representation that is eventually more meaningful. A simple analogy from medical practice is when one simplifies the age in years of a patient into a binary yes or no flag indicating whether a patient is above a critical age threshold. Here, we see a particular value for representation learning (eg, embeddings [31]) tailored to the unique characteristics of longitudinal health time series from clinical practice. Such AI solutions must be effective in dealing with the high-dimensional nature of medical data (clinical, genetic, social data, etc), avoid overfitting, and overcome the *curse of dimensionality* in the analysis. Mathematically, the idea of learning lower-dimensional representations is linked to manifold learning, for which embeddings are a special case [43].

Another challenge that further limits the use of AI-based trajectory analysis in medicine is data access and sharing. The current system is characterized by having data locked in *silos* where each hospital or health care institution limits access to their data and requires *lots of bureaucratic* work from researchers before allowing them to study and analyze the data. However, many initiatives are circumventing this status quo, such as the Observational Health Data Sciences and Informatics program, an international network of researchers aiming to provide reusable, collaborative, and reliable open-source solutions for large-scale health analytics [44]. Notably, there is interest in compiling extensive observational studies combining the electronic health record data from diverse health care organizations using standards to (1) design meaningful randomized controlled trials, (2) test clinical hypotheses on observational data, and (3) gain a better understanding of population characteristics, facilitated through framework efforts such as the Observational Health Data Sciences and Informatics program [44].

Furthermore, in the last couple of years, there has been an increasing number of studies focused on federated learning [45-47] that allows for AI algorithms to operate on decentralized data sets/systems in a privacy-preserving manner. In federated learning, the underlying algorithms (eg, FedAvg [48] and FedProx [49]) use the data stored in silos at different local sites for iteratively training a *global/central* model from a set of *local* models trained separately at each local site to perform prediction and classification tasks [48,50]. At the interface to health care, more research is being conducted that uses federated learning [45,51] for tasks such as clinical note phenotyping (ie, clinical natural language processing [52]) or predicting patient mortality in ICUs [53,54]. Here, we particularly point to the recent attempts to develop such approaches for patient trajectories. Examples include clustering patients through community-based federated machine learning for in-hospital mortality and length of stay prediction [55] or privacy-preserving patient similarity learning [56]. Federated learning may be further supported by secure hardware implementations, often with little computational overhead (eg, referred to as trusted execution environments [47]).

Moreover, there is more research on the privacy-preserving aspect of the technology, such as the differential privacy framework [57] (ie, applied to the model parameters), homomorphic encryption [58], and data anonymization techniques offering a *defensive level of privacy* as required by the General Data Protection Regulation and the Health Insurance Portability and Accountability Act [59]. Although these provide valuable tools for developers, we foresee more research that tailors them to the context of patient trajectories (eg, by offering sequential models for longitudinal data). More importantly, the availability of software packages [60,61] that allow both simulation of federated learning scenarios and their deployment in real clinical settings will accelerate the adoption of federated or distributed learning approaches and open a wide array of research exploration and experimentation possibilities. This will eventually yield longitudinal trajectory analyses that span patient journeys across multiple hospitals or health care institutions.

### Modeling

Fundamentally, analyzing patient trajectories requires AI solutions that can effectively handle sequential data structures that can vary in length (ie, from a few time points to multiple seconds, minutes, days, months, and year time windows). Hence, AI-based trajectory analysis must carefully adapt to the sequential structures by choosing appropriate modeling approaches.

In *risk scoring*, predictions from patient trajectories are often based on neural networks that are tailored to sequential data structures. These include tailored RNNs [3,17,27-29] owing to their strength in modeling long-term dependencies. One example of RNNs is the long short-term memory networks that iteratively process a time series with physiological measurements and aim to learn a lower-dimensional representation of the complete time series, regardless of its length, in their internal code layer. We can then use this lower-dimensional representation to predict patient outcomes from the patient’s trajectory. Gated recurrent units proceed analogously but have a more parsimonious structure that, in medical applications, may help in obtaining robust predictions (eg, with a lower risk of overfitting for small-sized data sets) [8,27,62]. Recently, digital medicine has also processed the patient trajectories through transformer networks [30]. Transformer networks involve attention layers that learn to weigh different parts in a patient trajectory differently while optimizing for the outcome prediction and may therefore outperform other RNNs. In our view, a particular benefit of transformer networks is that the developers from digital medicine can train them in using semisupervised learning. One can use a set of patient trajectories *without* observing the patient outcomes to learn an abstract representation (ie, via unsupervised pretraining). Subsequently, one can customize the transformer network to predict a specific patient outcome (ie, via supervised fine-tuning). Semisupervised learning often facilitates more efficient learning when the number of available observations with patient outcomes is comparatively low.

In risk scoring, other common prediction approaches are probabilistic models (eg, Markov models, point processes, and Gaussian processes) [23,32]. Here, we see several benefits for patient trajectory analyses in clinical settings. Probabilistic models often have a more parsimonious structure than the out-of-the-box neural networks, which facilitates efficient learning and reduces the risk of overfitting in data-scarce settings. In addition, such a parsimonious structure can facilitate interpretation by clinical practitioners. Probabilistic models can be naturally extended by latent structures (eg, hidden Markov models [63-67]), where latent states capture different trajectory phases and further improve interpretability. For instance, in the context of alcoholism treatment, patient trajectories have been modeled to undergo phases of *abstinence*, *moderate drinking*, and *heavy drinking*, each of which is captured by a separate latent state. In our view, such a latent structure represents a natural way to describe the different patterns in patient trajectories (eg, acute vs stable phases) and, more importantly, relates model characteristics to established clinical terminology. In the future, we expect hybrid models that combine the benefits of probabilistic modeling and neural learning (eg, deep Markov models [25]). The former benefits from theory-informed, interpretable structures that account for different disease states, whereas the latter are particularly effective for long-term dependencies.

**Figure 3:**
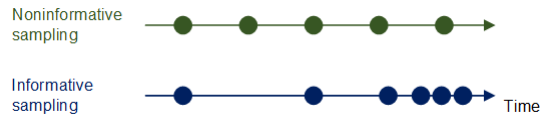
Difference between noninformative and informative sampling.

Across these risk models, it is further essential to consider the timing of the health measurement. Trajectories may consist of health recordings in equally spaced time intervals (eg, uniformly sampled every minute in ICUs or yearly intervals in patient registries). Often, they contain irregular time intervals, reflecting nonuniform and patient-specific interactions with the health care system. As a result, the sampling might be informative of the disease state (Figure 3). For instance, health professionals record more health measurements during deterioration in the patient’s health state. Therefore, AI solutions can use shorter time intervals to predict future decline in the trajectory. If the sampling is informative, we encourage researchers to develop models that consider the time intervals between the health measurements. For instance, 1 class of such models is point processes (eg, Hawkes processes). Here, a shorter time interval between health measurements makes further health measurements more likely and influences the expected risk score [23].

Figure 3 shows individual health recordings (eg, medical events, hospital visits, and laboratory data) in the form of dots. *Top:* an example patient trajectory where all medical events are equally spaced and thus there is a uniform time interval between the events. Here, the timing of the events is not informative of the current disease state. *Bottom:* an example patient trajectory that indicates a gradual decline in the disease state. Owing to this, additional health recordings are collected with higher frequency, which are thus informative about the disease state.

For objectives beyond risk scoring, we need other modeling approaches. When using risk scoring for *prescriptive purposes* (eg, treatment planning or dose finding), we encourage broader adoption of modeling strategies designed for decision making (eg, causal machine learning [37,68,69], Markov decision processes [70,71], dynamic treatment regimens [72,73], and policy learning [74]). There is a growing traction to extend many of these modeling strategies to handle longitudinal data, where health practitioners make treatment decisions over time.

For *subtyping*, one typically draws upon time-series clustering [26]. For this objective, the definition of a similarity metric is key [75,76]. One option is to set explicit rules for establishing patient trajectory similarities, for example, according to a specific condition such as heart disease [77] or a combination of clinical or phenotypic features [78]. Domain experts may be familiar with such approaches, but their definition may not perfectly integrate with AI algorithms and may thus require customization. Hence, an alternative is to view patient trajectory analysis from a methodological, *data-driven* angle. For example, when modeling the underlying temporal dynamics for performing data-driven clustering of longitudinal data, we can loosely group the approaches into (1) model-free approaches and (2) model-based approaches. In the model-free approaches, a similarity metric on sequential data is defined and then serves as input to conventional clustering algorithms. On the other hand, in the model-based approaches [79], a representation of the patient trajectory is learned, and the model parameters are then used for clustering (eg, mixture hidden Markov models). For future research, we find model-based approaches intriguing, as they no longer focus on raw observations but cluster the underlying disease dynamics (and, as such, can account for different latent disease states).

For *pathway discovery*, several descriptive approaches have emerged that allow for the discovery of subpatterns (ie, motifs) and common patient trajectories [76]. For example, Beck et al [33] analyzed disease pathways leading to septicemia in 110,000 patients. The study revealed prototypical pathways starting from 3 initial states (alcohol abuse, diabetes, and anemia) and established the trajectory-specific probability of sepsis mortality. This and similar studies reveal great potential to further our understanding of disease etiology and the possible means of changing disease trajectories [80,81]. Similarly, Oh et al [82] constructed patient trajectories consisting of specific health events (hyperlipidemia, hypertension, and impaired fasting glucose) and evaluated the probabilities of such trajectories (and their permutation) in increasing or decreasing the log odds of developing type 2 diabetes mellitus. Zhang and Padman [83] identified the most probable clinical trajectories from patients with chronic kidney disease by first grouping the patients and then fitting a first-order hidden Markov model to infer the most probable clinical pathways given sequences of multiple laboratory test observations and other patient characteristics. Huang et al [84] proposed a probabilistic model (based on latent Dirichlet allocation) to identify clinical pathway patterns from the event logs for patients with unstable angina and cancer. Dabek and Caban [85] offered another perspective by analyzing trajectories using automata (ie, deterministic and nondeterministic finite state automata) and using a grammar induction algorithm to identify common trajectories in neurology. Further approaches for pathway discovery are based on association rule mining [86] and functional dependencies mining [87].

Related to these objectives are models that adopt a structural lens to examine the causal mechanisms [88]. This would allow not only to understand *how* health measurements change over time but also *wh*y. The underlying AI algorithms are still under active research (eg, causal structure learning and neural causal discovery [89]). Here, it will be a rewarding direction for the future to develop more approaches that are tailored to longitudinal data.

### Evaluation

Evaluations through randomized controlled trials are needed to confirm the effectiveness of AI-based analysis of patient trajectories in clinical practice. Recently, there have been such trials for traditional AI solutions that rely on snapshot data from a single or few time points [90]. However, similar trials for patient trajectory analysis are rare [90]. We expect significant value in conducting such trials and foresee challenges owing to the unique characteristics of patient trajectories. Foremost, evaluations through rigorous randomized controlled trials are a prerequisite to building trust in clinical practice, thereby expediting further AI-based trajectory analysis. However, evaluating such an AI solution might be a multiyear undertaking depending on the time window of the patient’s trajectory. Thus, it is also essential to recognize that the evaluations are likely to involve a 2-step procedure. In the first step, trajectory data are collected to train the AI solution. In the second step, the previously trained AI solution is then deployed to analyze how the AI solution generalizes to new patient trajectories. When conducting such trials, it is crucial to acknowledge that the patient trajectories capture data from multiple time points and might thus be subject to an inherent domain shift (ie, where data distributions change over time, that is, over the patient journey) [91]. Such domain shifts might affect the performance of AI solutions [92], especially when the patient trajectories span a long time horizon. For example, AI-based predictions have been recently applied to patients with COVID-19 to compare the predicted health trajectory with the observed trajectory in a prospective study, finding that the performance of some risk scores decreased over time [93]. One reason was because of temporal domain shifts over time [94] as medical professionals learned about the emerging infectious disease and adapted their clinical routines over time, thus yielding different and, in particular, better outcomes than in the data used for training.

Furthermore, there is a need to ensure reproducibility of the AI-based analyses. Here, we consider 3 priorities. First, there is a need to develop a framework. For instance, the existing AI frameworks (such as scikit learn in Python) are designed for modeling static data. Conversely, more effort is necessary to define standardized building blocks for longitudinal data to effectively model the patient trajectories. Moreover, such frameworks should also involve tools for automation so that the disease models can be trained in a semiautomated manner (subsumed under the term *automated machine learning* [*AutoML*]). Although AutoML has become widespread for static data sets, only a few libraries are designed for time-series AutoML [95]. Here, we see enormous potential for future research at the interface to digital medicine. On the basis of our own experience, we expect such frameworks to play a critical role in achieving scalable and increased adoption of AI-based patient trajectory analysis in clinical settings. Second, future research should carefully assess and, if needed, revise the best practice guidelines for AI in medicine [96], so that they consider the longitudinal form of patient trajectories. For example, the reporting guidelines should involve information on whether the period between health measurements is informative (however, such information is not part of existing reporting guidelines for static patient data). Other examples could be the choice of the model (eg, whether latent dynamics were considered and why or which alternative architectures of recurrent neural networks were tested and eventually discarded), how the timing of the health care event was collected (eg, whether a time stamp was retrieved from a medical health record or whether this was a survey question referring to the *last 12 months* and the resulting uncertainty about the correct time), or the rationale behind how patient similarity was measured in subtyping. Finally, more data sets with patient trajectories should be made publicly available for benchmarking. Although data access is a common issue for AI research in medicine in general, the challenges are exacerbated in the context of patient journeys, where it is common to merge the health measurements from different sources (eg, from other health registries). So far, only a few longitudinal data sets are public (as compared with static data sets with patient data) [76]. Notable exceptions are large initiatives, such as the Healthcare Cost and Utilization Project, that offer longitudinal data sets for evaluating trajectory-based AI approaches [97].

### Interpretations

To generate insights for clinical practice, it is often necessary that AI solutions overcome their black-box nature [98]. Here, we see enormous potential for new AI solutions that adhere to the needs of clinical practice with the objective of knowledge discovery.

One approach is explainability, which aims to understand how a model arrives at a particular outcome [10,99]. However, AI explainability is typically developed in the context of static data and therefore, meaningful time-varying patterns from the course of a disease might not be revealed. For instance, SHAP values [99] inform which health measurements are used by the AI model and what values indicate risk. However, SHAP values cannot directly interpret the *dynamics* in health measurements (eg, whether there is an increase or decrease or large variability in health measurements, which would be needed to characterize changes in disease states over time). As a road map for research in digital medicine, we require techniques that interpret the temporal dynamics of disease progression, thereby being closely aligned with the demands of clinical practice. Here, digital medicine might find inspiration in other disciplines, for instance, financial technical analysis [100], where a systematic set of short-term movements of stock prices is used for interpretation. Similar patterns in patient trajectories could be inferred by researchers via short-term patterns (ie, trajectory markers) that characterize a disease course or a combination of short- and long-term motifs that help identify distinct disease states.

Another approach to generating insights is via interpretability, which builds upon inferences where the underlying logic is transparent. Interpretability often requires tailored modeling approaches. For static data, this is usually achieved through (penalized) linear regression or decision trees, whereas interpretability for longitudinal data is typically achieved through parsimonious models. Different strategies exist to obtain parsimonious models. For neural networks, there are techniques that tweak a neural network to provide a sparse one with similar performance (eg, enforcing feature sparsity through architecture design, modifying the objective function and the weight updating scheme [101], post hoc via reservoir computing or pruning, or a priori via cognitive networks [102]). Alternatively, one can draw upon structural formalizations (eg, dynamic fuzzy cognitive maps [78] to simulate patient trajectories) and probabilistic models (eg, Markov models, hidden Markov models, and Hawkes processes [23,25,63-67]).

**Figure 4:**
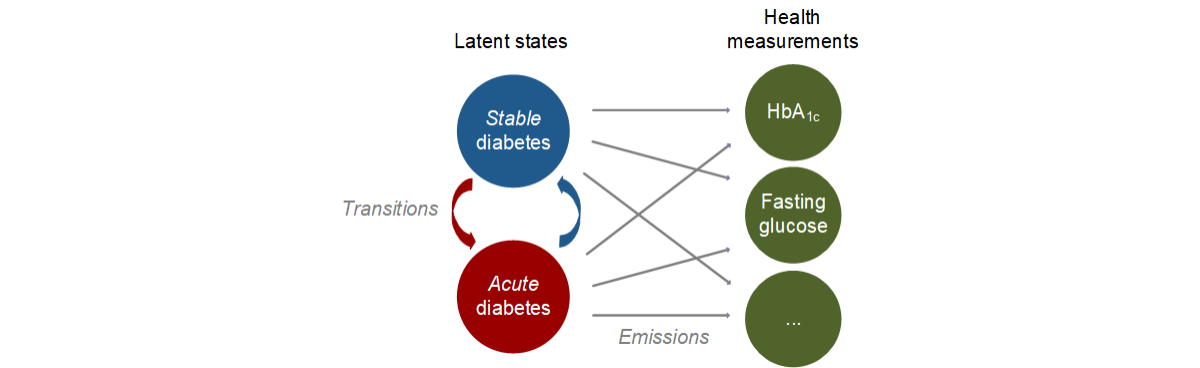
Example of a hidden Markov model. HbA1c: hemoglobin A1c.

Out of these modeling approaches, we expect hidden Markov models to be beneficial for interpretability, especially for risk scoring. The reason being hidden Markov models use latent variables to capture different disease phases in patient trajectories. These latent variables often have clinically relevant meanings and can thus be mapped onto existing clinical terminology (Figure 4). For instance, in diabetes mellitus, the latent states are characterized as *acute* and *stable* disease states [64] and thus are of clinical meaning. In addition, recent evidence suggests that interpretable models might also improve prediction performance [23]; however, more effort is needed to explore this further. In the future, we expect to see other modeling approaches that combine the strengths of hidden Markov models (for interpretability) with neural learning (for representation learning and capturing long-term dependencies in patient trajectories).

The health measurements are observable and thus obtained via standard data collection practices. In contrast, the latent states cannot be observed directly and, instead, are recovered from the health measurements. The latent states then describe different disease states in a patient trajectory (eg, *acute* vs *stable* disease states). During estimation, the latent states and health measurements are mathematically linked via components for both transition and emission probabilities.

## Implications for Digital Medicine

Analyzing patient trajectories using AI has multiple benefits. Dissecting the variability of disease pathways allows research to better understand both the disease etiology and disease course, facilitating a more extensive personalization of care. For instance, it enables the identification of short-term patterns predictive of future health states, which are then used during risk scoring. Similarly, patient trajectories capture the responsiveness of patients to treatments, and by leveraging this information in patient trajectories, AI solutions can guide treatment planning. In summary, AI-based trajectory analysis promises to strengthen the existing computational approaches to prevent, detect, diagnose, and treat diseases.

To address these challenges, we see particular importance in community building and in the development of computational patient trajectory tools that lower the barrier of entry. Community building will help to set a clear agenda and define an established terminology, bridging both practice and research in digital medicine. Here, we point to several valuable directions: (1) further effort will be needed to extend traditional clinical terminology (eg, cohort building and patient similarity) to AI-based trajectory analysis, thereby facilitating communication between the experts in AI and medicine; (2) it is essential to build communities by connecting different actors from regulation, law, data science, and medicine, as this will eventually be a prerequisite for deploying AI solutions in medical practice; and (3) such communities may promote data exchange, thus allowing for more extensive benchmarking of AI solutions. Similarly, we also suggest hosting leaderboard competitions as conducted in other fields (eg, the SemEval benchmark competitions in natural language processing). Leaderboard competitions will eventually help to identify robust AI solutions and thus to condense best practices during modeling.

## Abbreviations

AI: artificial intelligence
AutoML: automated machine learning
ICU: intensive care unit
RNN: recurrent neural network
